# Dramatic Response of a Large Sacral Chordoma to Intensity Modulated Proton Beam Therapy

**DOI:** 10.7759/cureus.1670

**Published:** 2017-09-10

**Authors:** Craig Schneider, Melissa Vyfhuis, Emily Morse, Tejan Diwanji, James W Snider, Sina Mossahebi, Katarina Steacy, Robert Malyapa

**Affiliations:** 1 Radiation Oncology, University of Maryland School of Medicine

**Keywords:** sacral chordoma, chordoma, intensity modulated proton therapy (impt), pencil beam scanning proton therapy

## Abstract

Sacral chordomas are slow-growing, indolent, and locally invasive tumors that typically present with pain and neurologic dysfunction. Wide en-bloc surgical excision is the primary treatment, but achieving adequate margins is difficult and surgery is often associated with significant morbidity. Adjuvant radiation therapy (RT) is utilized to decrease the risk of local recurrence or as definitive treatment for nonsurgical candidates. Although chordomas are considered to be relatively radioresistant tumors, several studies have demonstrated tumor response to high-dose proton therapy. Here, we present a patient with a large sacral chordoma who underwent definitive treatment with intensity-modulated proton therapy (IMPT).

## Introduction

Chordomas are a rare form of bone cancer that is derived from undifferentiated notochordal remnants, with an incidence of approximately 0·08 per 100,000 [1]. Chordomas are predominantly found in men, with a peak occurrence between 50–60 years of age. Despite a low tendency to metastasize, clinical outcomes remain poor, with a median survival of 6.3 years. As a consequence of their origin, chordomas may arise anywhere along the neural axis but are most commonly found in the sacrococcygeal region (30%-50%) and the skull base [2]. Sacral chordomas are indolent, locally aggressive, and invasive tumors, which often remain clinically silent until they become locally destructive, resulting in pain and neuropathies [1,3].

The management of these tumors is particularly challenging. Chordomas often have a large tumor burden at the time of diagnosis and tend to grow along critical bony and neural structures, making surgical resection especially difficult and morbid. En bloc resection is only possible in approximately half of the cases of sacral chordomas [3]; therefore, adjuvant radiation therapy (RT) is commonly recommended to improve local control. However, chordomas are relatively resistant to conventional photon-based RT, which even in the adjuvant setting, is associated with high rates of local recurrences [1]. Recent studies have demonstrated greater local control rates and improved tolerance to treatment with intensity modulated proton therapy (IMPT), providing evidence for a more effective alternative to traditional photon RT in the treatment of sacral chordomas [4-5].

## Case presentation

A 79-year-old Caucasian man with no significant past medical history presented for the evaluation of a previously diagnosed sacral chordoma. The tumor was incidentally found five years prior after imaging studies were performed to evaluate the injuries sustained after a motor vehicle accident. The mass was biopsied two years after the initial discovery, and pathology confirmed the diagnosis. At that time, surgical excision was recommended but the patient declined surgery due to fear of post-surgical morbidities.

**Figure 1:**
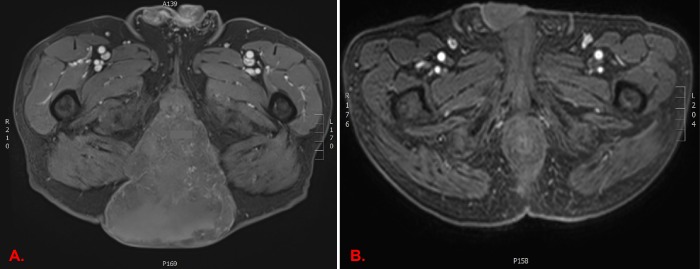
Axial T1 post-contrast MRI scan one year before (A) and nine months after (B) proton beam therapy with a complete radiographic response to treatment. MRI: Magnetic resonance imaging

**Figure 2:**
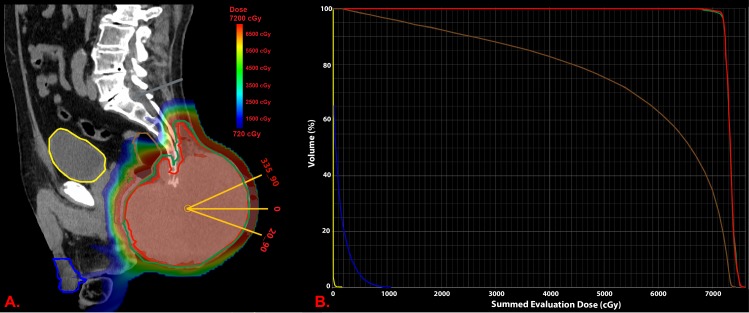
Sagittal view of pencil beam scanning proton treatment plan (A) and dose-volume histogram of the prescribed 72 Gy dose (B). A). Three posterior beams were utilized at 335, 20, and 0 with the first two delivered in a noncoplanar fashion with a 90° degree couch kick. The bladder is outlined in yellow, rectum in brown, and external genitalia in blue. Gross tumor volume (GTV) is in red and clinical tumor volume (CTV) is in green. B). Dose-volume histogram (DVH) of prescribed 72 Gy dose, with GTV in red, CTV in green, bladder in yellow, rectum in brown, and external genitalia in blue.

**Figure 3:**
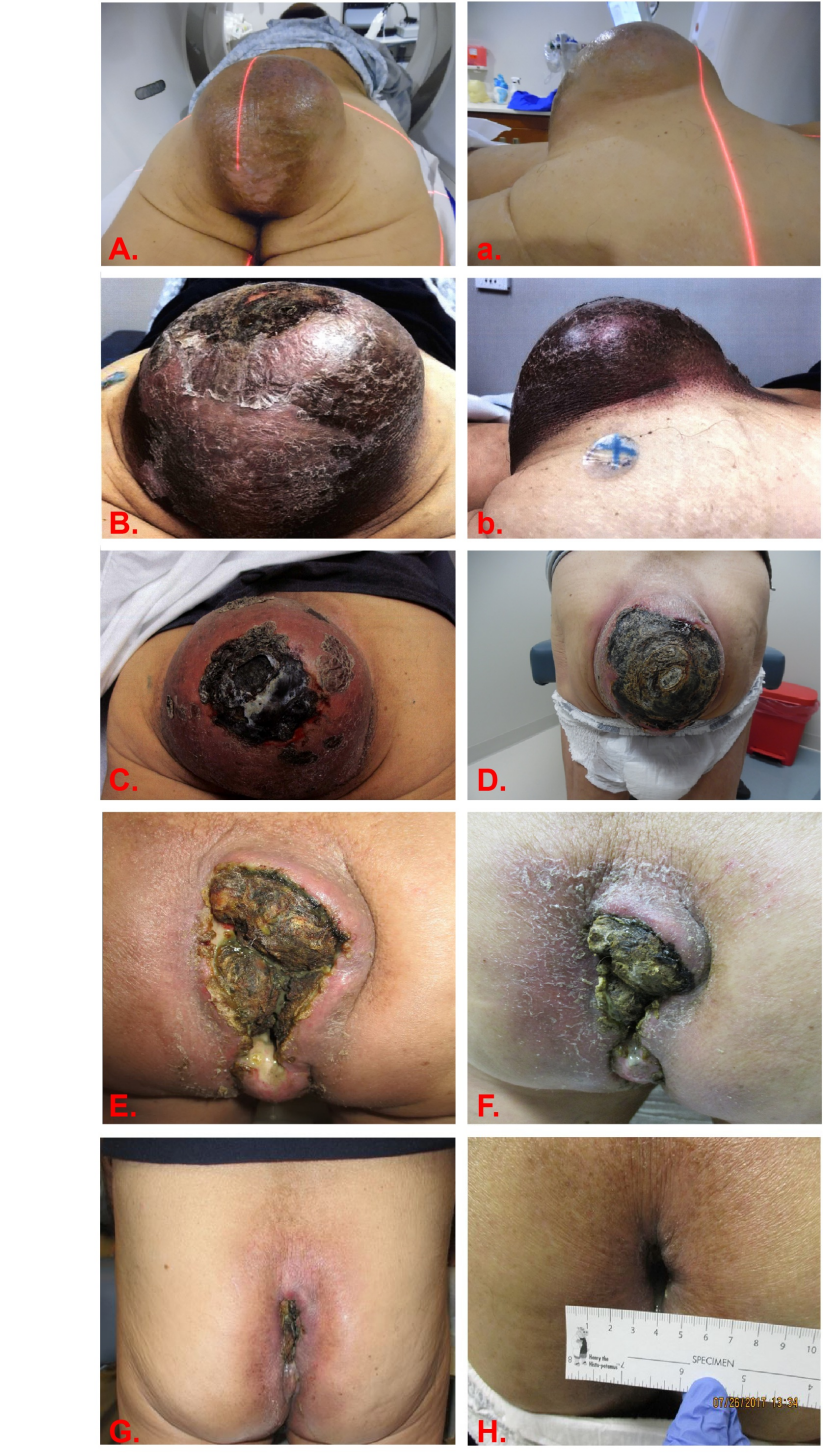
Clinical response of sacral chordoma to PBS-PT. Axial and sagittal view of the sacral chordoma before (A, a, respectively) and immediately after (B, b, respectively) treatment. C.) one month after treatment; D.) three months after treatment; E.) four months after treatment; F.) five months after treatment; G.) six months after treatment; and H.) and 8 months after completing proton therapy. PBS-PT: Pencil beam scanning proton therapy

The patient's most recent magnetic resonance imaging (MRI) scan performed two months prior to presentation showed a 15.5 cm x 14.8 cm x 15.6 cm mass with thinning and extensive involvement of the posterior pelvic floor overlying the subcutis with a small presacral component (Figure 1A). A computed tomography (CT) scan of the chest, abdomen, and pelvis was also performed at this time and revealed no distant metastatic disease. At presentation, the patient reported pain in the sacral spine when sitting, but denied any neurologic sequelae. He continued to jog three miles daily without difficulty but was not able to comfortably sit for any period of time. The physical exam was unremarkable with the exception of a large, protruding mass in the sacral area, approximately 15 cm in size (Figure 1A, Figure 2A, Figure 3A, Figure 3a). Due to the patient’s desire to avoid surgical resection, he was offered definitive proton irradiation.

Radiation treatment

The patient underwent CT simulation in the prone position, arms up, with a Vac-Lok bag (CIVCO Radiotherapy, Coralville, Iowa) used for immobilization. The gross tumor volume (GTV; red in Figure 2A) was contoured using the CT simulation scan and a 5 mm expansion was added to the GTV for the clinical target volume (CTV; green in Figure 2A), excluding the rectum and staying within 3 mm from the skin. We prescribed a total dose of 72 Gy given in 2.25 Gy per fraction. We delivered his RT using pencil beam scanning proton therapy (PBS-PT). Three posterior-anterior (PA) fields were positioned at 0°, 20°, and 335°; the latter two fields were delivered via a non-coplanar approach utilizing a 90°-couch kick (Figure 2A). A single field optimization (SFO) plan was carried out using 3.5% range uncertainty and 5 mm set-up uncertainty in the principle anatomical planes as previously described [6]. We used daily X-rays for image guidance as well as weekly quality assurance (QA) CTs to assess for soft-tissue changes in the primary tumor and rectal filling. The final plan was acceptably robust, where 95% of the CTV was receiving greater than 95% of the prescribed dose. The bladder (yellow in Figure 2A) and external genitalia (blue in Figure 2A) received a maximum dose of 1.4 and 10.5 Gy, respectively. The V70 of the rectum (the percent of rectal volume receiving 70 Gy), outlined in brown in Figure 2A, was 16%, and the V65 (the percent of rectal volume receiving 65 Gy) was 47%, with a maximum point dose of 71.7 Gy with a total dose delivered of 69.75 Gy. The dose-volume-histogram (DVH) of prescribed dose is summarized in Figure 2B. 

Response to proton therapy

WB tolerated his treatment fairly well. By the end of his radiation course, he started having significant discomfort at his tumor site, especially with ambulation. He did not have urinary or rectal side effects but did experience grade 3 pain, as well as grade 2 fatigue and radiation dermatitis (National Cancer Institute (NCI) Common Terminology Criteria for Adverse Events (CTCAE) V4.03 toxicity grading) by the end of treatment (Figure 3B and Figure 3b). He did not receive his last fraction due to his inability to sit in a car to travel for treatment, therefore, he received only 31 out of 32 planned treatments (69.75 Gy). Eight months after treatment, his pain has been slowly improving to the point where he is ambulating comfortably. He continues to have no rectal symptoms and is having regular bowel movements. He also reports a significant improvement in his quality of life, as he is able to utilize a regular commode for defecation, which he was unable to tolerate previously due to the mass. Treatment produced a drastic radiographic (Figure 1B) and clinical response with gradual necrosis and sloughing of large portions of the tumor following completion of treatment (Figure 3A-H).

 

## Discussion

The standard approach to the treatment of chordomas is maximal, safe surgical resection followed by adjuvant proton therapy, as complete surgical excision is often not feasible due to tumor invasion into local structures. Studies suggest that high dose RT (> 70 Gy radiobiological equivalents or GyRBE) is efficacious both as an adjuvant to surgery and as definitive therapy [4-5]. However, the delivery of high dose photon RT to chordomas is problematic due to dose-limiting organs-at-risk (OARs). For sacral chordomas, important nearby structures at risk of off-target radiation toxicity include the spinal cord, sacral nerve roots, bowel, bladder, and ovaries. Particle-based RT, such as proton or carbon ion therapy, intrinsically deposit relatively low doses, as they travel through tissue before depositing a high dose right before they come to rest at a set depth in the tissue with no exit dose. This phenomenon, called the Bragg Peak, can be exploited to deliver high doses of particle-based RT to the target while minimizing dose to nearby tissue. For this reason, proton therapy has been utilized in place of, or in combination with, traditional photon RT to allow the delivery of high dose RT to chordomas while minimizing toxicity.

Definitive high dose proton RT is recommended for patients with sacral chordomas that are deemed poor surgical candidates or in patients that decline surgery. Surgical resection can result in significant morbidities, which include sexual dysfunction and bowel and urinary incontinence [3]. Data on the treatment of sacral chordomas with definitive, high dose proton RT is sparse, but a handful of retrospective analyses have demonstrated safety and efficacy [4-5,7]. A 2014 study out of Japan retrospectively evaluated the efficacy of definitive proton or carbon ion RT on 23 patients (seven receiving proton therapy) with sacral chordomas treated to 70.4 GyRBE in either 16 (4.4 GyRBE/fx) or 32 fractions (2.2 GyRBE/fx) [5]. With a median follow-up of 38 months, three-year local control, overall survival (OS), and progression-free survival (PFS) were reported to be 94%, 83%, and 68%, respectively. There was no statistically significant difference in local control or OS between either heavy-ion use.  

A more recent study from Mass General Hospital (MGH) provided an update on the outcomes and responses of spine and sacral chordomas treated with definitive photon and/or proton RT (all but one patient received proton RT) [4]. A total of 40 patients with spine (32.5% of patients) or sacral chordomas (67.5% of patients) receiving 77.4 GyRBE resulted in five-year local control, OS, and disease-specific survival of 85.4%, 81.9%, and 89.4%, respectively. The authors also reported a significant reduction in the total target volume over time with an average reduction of 55% and 65% at two and five years’ post-treatment, respectively.

In the case presented here, the patient experienced a dramatic response following definitive proton RT to 69.75 Gy (76.7 GyRBE) using PBS-PT, despite his extensive disease at presentation (Figure 1A). Several studies evaluating chordoma response to RT have suggested that size correlates to poor prognosis chordomas after high-dose RT [4-5,8]. As of yet, only one institution has published their experience with PBS-PT for the treatment of sacral chordomas [7]. Researchers at the Paul Scherrer Institute (PSI) analyzed the outcome of 102 patients with spinal chordomas (37% sacral chordomas) treated with high-dose PBS-PT immediately following surgical resection (70%) or after local recurrence (30%). The median RT dose delivered was 73.8 GyRBE with the vast majority of patients (87%) receiving exclusively PBS-PT (13% received combined photon-proton RT). The authors reported the five-year LC, OS, and PFS were 64.9%, 59.4%, and 80.5%, respectively, with minimal high-grade toxicities. Grade 3 or higher toxicities were 8% and 9% for acute and late events, respectively.

High doses of RT are necessary to achieve reasonable clinical outcomes in sacral chordomas, which can, unfortunately, result in treatment side effects. Radiation-induced dermatitis and pain are the most commonly reported adverse events in patients with sacral chordomas receiving high-dose proton RT (Figure 2B and Figure 2b) [4-5]. As reported in the Japanese study by Mima et al., a significant fraction of patients experienced radiation-induced dermatitis (35% of patients) with fewer patients experiencing late neuropathies (17%) and myositis (9%) [5]. However, the majority of treatment-related toxicities were observed in the 16 fraction group (4.4 Gy/fx), accounting for 89% and 82% of acute and late reported adverse events, respectively. Patients receiving 32 fractions (2.2 Gy/fx) were less likely to have treatment-related side effects. Despite these adverse events, all patients in this study were able to complete their treatments as planned and recovered from their acute reactions.

Similarly, in the MGH experience, the most common acute side effects reported were radiation-induced dermatitis (grades 1 to 2) and pain [4]. Grade 1-2 nausea and vomiting (10% of patients), mucositis (12.5% of patients), and diarrhea (12.5% of patients) were also observed. Reported late toxicities included sacral insufficiency fractures (25% of patients, none required surgical stabilization), various manifestations of sacral neuropathies (15% of patients), and bowel perforation/fistula formation (one patient). Other complications observed following high-dose proton RT include reports of secondary malignancies, sacral insufficiency fractures, soft tissue necrosis/deformity, erectile dysfunction, and rectal bleeding [8-10].

## Conclusions

While wide en bloc resection of sacral chordomas with or without adjuvant RT remains the standard of care, definitive treatment with high-dose proton RT using the PBS technology is a safe alternative for patients that are unable to undergo, or decline, surgical resection. Definitive high-dose proton RT provides excellent five-year local control and overall survival rates with a reasonable toxicity profile. Long-term complications, such as sacral neuropathies, are less commonly observed.

## References

[1] Walcott BP, Nahed BV, Mohyeldin A, Coumans JV, Kahle KT, Ferreira MJ (2012). Chordoma: current concepts, management, and future directions. Lancet Oncol.

[2] McMaster ML, Goldstein AM, Bromley CM, Ishibe N, Parry DM (2001). Chordoma: incidence and survival patterns in the United States, 1973-1995. Cancer Causes Control.

[3] Fuchs B, Dickey ID, Yaszemski MJ, Inwards CY, Sim FH (2005). Operative management of sacral chordoma. J Bone Joint Surg Am.

[4] Kabolizadeh P, Chen YL, Liebsch N (2017). Updated outcome and analysis of tumor response in mobile spine and sacral chordoma treated with definitive high-dose photon/proton radiation therapy. Int J Radiat Oncol Biol Phys.

[5] Mima M, Demizu Y, Jin D (2014). Particle therapy using carbon ions or protons as a definitive therapy for patients with primary sacral chordoma. Br J Radiol.

[6] Malyapa R, Lowe M, Bolsi A, Lomax AJ, Weber DC, Albertini F (2016). Evaluation of robustness to setup and range uncertainties for head and neck patients treated with pencil beam scanning proton therapy. Int J Radiat Oncol Biol Phys.

[7] Snider JW, III III, Stieb S, Poelma-Tap D (2016). Long-term outcomes following pencil beam scanning proton radiation therapy for spinal chordomas: a large, single-institution cohort. Int J Radiat Oncol Biol Phys.

[8] Demizu Y, Mizumoto M, Onoe T (2017). Proton beam therapy for bone sarcomas of the skull base and spine: A retrospective nationwide multicenter study in Japan. Cancer Sci.

[9] Rotondo RL, Folkert W, Liebsch NJ (2015). High-dose proton-based radiation therapy in the management of spine chordomas: outcomes and clinicopathological prognostic factors. J Neurosurg Spine.

[10] DeLaney TF, Liebsch NJ, Pedlow FX (2014). Long-term results of Phase II study of high dose photon/proton radiotherapy in the management of spine chordomas, chondrosarcomas, and other sarcomas. J Surg Oncol.

